# Neglected diseases of neglected populations: Thinking to reshape the determinants of health in Latin America and the Caribbean

**DOI:** 10.1186/1471-2458-5-119

**Published:** 2005-11-11

**Authors:** John P Ehrenberg, Steven K Ault

**Affiliations:** 1 Chief, Communicable Diseases Unit, Area of Disease Prevention and Control, Pan American Health Organization/World Health Organization (PAHO/WHO), 525 23rd Street NW, Washington, DC 20037, USA; 2 Regional Advisor, Communicable Diseases Unit, Area of Disease Prevention and Control, Pan American Health Organization/World Health Organization (PAHO/WHO), 525 23rd Street NW, Washington, DC 20037, USA

## Abstract

**Background:**

People living in poverty throughout the developing world are heavily burdened with neglected communicable diseases and often marginalized by the health sector. These diseases are currently referred to as *Neglected Diseases of Neglected Populations*. The neglected diseases create social and financial burdens to the individual, the family, the community, and the nation.

**Discussion:**

Numerous studies of successful individual interventions to manage communicable disease determinants in various types of communities have been published, but few have applied multiple interventions in an integrated, coordinated manner. We have identified a series of successful interventions and developed three hypothetical scenarios where such interventions could be applied in an integrated, multi-disease, inter-programmatic, and/or inter-sectoral approach for prevention and control of neglected diseases in three different populations: a slum, an indigenous community, and a city with a mix of populations.

**Summary:**

The objective of this paper is to identify new opportunities to address neglected diseases, improve community health and promote sustainable development in neglected populations by highlighting examples of key risk and protective factors for neglected diseases which can be managed and implemented through multi-disease-based, integrated, inter-programmatic, and/or inter-sectoral approaches. Based on a literature review, analysis and development of scenarios we visualize how multiple interventions could manage multiple disease problems and propose these as possible strategies to be tested. We seek to stimulate intra- and inter-sectoral dialogue which will help in the construction of new strategies for neglected diseases (particularly for the parasitic diseases) which could benefit the poor and marginalized based on the principle of sustainability and understanding of key determinants of health, and lead to the establishment of pilot projects and activities which can contribute to the achievement of the Millennium Development Goals.

## Background

People living in poverty throughout the developing world are heavily burdened from a series of communicable diseases, particularly parasitic diseases. They also tend to be marginalized by the health sector, as are many of the diseases that affect them. These diseases which are currently referred to as *Neglected Diseases of Neglected Populations*, pose a major challenge to the fulfillment of the Millennium Development Goals [1]. Some of these neglected diseases (NDs) of parasitic origin include lymphatic filariasis, soil-transmitted helminthiasis, schistosomiasis, onchocerciasis, leishmaniasis, African trypanosomiasis, Chagas disease, ectoparasitic skin infestations and parasitic zoonoses, among others [2].

These diseases are called neglected because they affect the poor, they are not subject to compulsory reporting in most countries, and are therefore not perceived as major public health burdens as compared to HIV/AIDS, tuberculosis, and malaria for instance. Most of them do not lead to epidemiologic emergencies, and consequently attract little attention from the media and the public sector. Furthermore, the private sector does not necessarily consider this group of diseases as a lucrative target, a phenomenon which severely hampers spending on research and development of specific drugs, vaccines and diagnostic tools [3]. However, new non-profit drug companies and private-public partnerships are beginning to address this gap at least for leishmaniasis, African trypanosomiasis and Chagas disease [4].

### Burden of disease and vulnerable groups

The neglected parasitic diseases result in a high financial burden to the individual, the family, the community, the country and even the region – impairing its development [5]. For example, in the late 1970s, US$4.4 million were lost annually by people infected with *Ascaris lumbricoides *in Kenya, just in the form of unabsorbed food [6], and such costs can only rise as a population grows in the absence of specific prevention and control measures. A recent meta-analysis reassessing the disabilities created by schistosomiasis infections has concluded that this disease causes significantly more *accumulated *morbidity (anemia, chronic diarrhea and pain, undernutrition from protein loss, exercise intolerance, infertility, poor school performance) than previously thought, indicating it creates a very significant burden on the health of infected individuals [7]. Parasitic diseases, whether vector-borne, food-borne, water-borne or soil-transmitted tend to affect certain vulnerable groups, such as school-age children, the women of childbearing age or the breadwinners (male or female) in a household [8]. For example, poor working people with chronic lymphatic filariasis in Orissa, India lose an average of 68 workdays per year (19% of their work year), and spend an average of US$8.70 per year for treatment of their condition [9], much more than the per capita health expenditure by the national health services. Other vulnerable groups in society such as indigenous populations and minority ethnic groups, infants and pre-school children, the elderly, those with physical limitations, and immune-compromised people such as those with HIV/AIDS can be highly burdened with certain parasitic and other communicable diseases. Additional high-risk populations often include people living in slums [10], migrant workers (e.g., itinerant gold miners in Brazil [11]) and those living in agricultural labor camps or plantations (e.g., Guatemalan coffee pickers with onchocerciasis [12]).

### Objective

The objective of this paper is to identify new opportunities to address NDs, improve community health and promote sustainable development in neglected populations by highlighting examples of key risk and protective factors of NDs which can be managed through *multi-disease-based, integrated, inter-programmatic, and/or inter-sectoral approaches*.

Many determinants of health lie *outside *the purview of the health sector. Furthermore, the policies of the sectors that exert influence on these negative health impacts are usually not established according to public health criteria (see Section A, below). Consequently, addressing comprehensive and sustainable solutions to these health problems cannot be the sole responsibility of the health sector. Soil-transmitted helminthiasis and schistosomiasis are good examples of the multi-sectoral and multi-factoral contextual determinants of NDs, where interventions via other sectors to improve water quality and quantity, provide safe excreta disposal, combined with periodic drug treatment and health education are the keys to sustainable control [13,14].

Partnerships with other sectors capable of effective action will be necessary. The reduction of NDs will ultimately contribute to the sustainable development of poverty stricken populations and may contribute to an increase in economic growth of the countries affected by these diseases.

## Discussion

### Rationale

Every day thousands of people living in poverty get sick and suffer or die of preventable, communicable diseases throughout the world. This accounts for the major difference in the magnitude of mortality and morbidity rates between developed and developing countries. Among the communicable diseases, the NDs such as lymphatic filariasis may be considered proxy indicators of the level of socioeconomic development [15], and communicable diseases are pervasive in countries or regions where gross national product is low or income distribution highly skewed [16]. Some of these diseases would cease to exist with an increase of the gross national product and a more balanced income distribution. However, this is a long-term solution to problems that demand immediate actions.

NDs inhibit the capacity of poor and neglected communities to achieve sustainable development, that is – the physical disability, stunting of child growth and intellect, and mortality created by NDs weakens the ability of both current and future generations to meet their basic human needs in a long-term manner, generate sufficient income, and may also constrain them from innovating and adopting ecologically sustainable practices at home and work.

This proposed strategy for the prevention and control of NDs in neglected populations is based on integrated, multi-disease, inter-programmatic and/or inter-sectoral approaches to manage multiple health risks and protective factors in the short and medium term, similar to that recently suggested by Molyneux and Nantulya [17] and others [18,19]. For example, in view of the American region's current demographics, provisions will need to be taken to tailor the approaches to both urban and rural populations (70% of the population in the Americas is urban). The expectation is that this new set of approaches can increase program sustainability of NDs control and elimination efforts. Furthermore, this set of approaches could strengthen existing health services and epidemiologic surveillance systems [20], as well as contribute to their integration into a multi-disease based surveillance and control system.

### A. Multifactorial determinants of disease

In order to successfully develop an agenda for NDs in neglected populations, it is important to consider the contextual determinants of health. These are both intrinsic and extrinsic to human populations and their combination will determine the epidemiological pattern of these communicable diseases (disease spectrum).

#### Intrinsic determinants

Intrinsic determinants of disease are biological in nature (i.e., genetic makeup, immune response). Most of the intrinsic determinants can be manipulated only as a function of advances in medical research and technology (e.g., development of new vaccines, drugs and diagnostic tools). Significant progress has been made (private and academic sectors) in developing some of these tools, specifically those that target lucrative markets. However, their development for tropical diseases (e.g., the Tropical Diseases Research/World Health Organization drug research program and private sector initiatives) has been slow and it has been very difficult for neglected populations to access these tools given their high costs. This is clearly an issue of inequity in health services delivery to the poor that deserves further attention.

#### Extrinsic determinants

Extrinsic determinants of disease include poverty, vector ecology and behavior, and various human activities (sometimes combined with natural disasters) such as poorly planned agricultural and irrigation development, uncontrolled urbanization, and indiscriminant insecticide use, and improper self-treatment with medications. These are discussed below.

#### Poverty

Clearly, poverty is one of the most critical extrinsic determinants that impact the health of *individuals *and *groups*. It also increases the vulnerability to diseases by limiting their access to high quality health care, good housing and safe food [21]. It is also associated with social violence [22], drug addiction [23] and HIV/AIDS transmission [24]. Managing these determinants would require intense advocacy, improving living conditions, implementing health and environmental education, and social communication.

#### Vector ecology and behavior

Extrinsic determinants also include vector behavior, as well as characteristics of their habitat or environments [25]. The World Health Organization (WHO) has recently developed a strategic framework for Integrated Vector Management (IVM) to improve the control of malaria, dengue, and Chagas disease among other vector-borne diseases [26] with emphasis on interventions based on vector ecology and environmental determinants. Furthermore, some communities are currently experimenting with incorporating IVM into their on-going vector-borne disease control programs (e.g., in lymphatic filariasis elimination programs; and in malaria, leishmaniasis and schistosomiasis and Chagas disease control programs).

#### Human activities and the environment

Another group of extrinsic determinants of health include human activities and environmental determinants [27]. Human activities have an impact on the environment, and in doing so, they create conditions which have an impact on the epidemiological pattern of some communicable diseases including NDs [25]. They may also increase susceptibility to natural disasters (e.g., intense deforestation combined with heavy rains in Haiti led to disastrous mud slides in 2004). Some natural disasters such as flooding can lead to more breeding sites for disease vectors [28], and to an increased risk of disease transmission and outbreaks [29]. The indiscriminate use of insecticides in agriculture and public health interventions has led to resistance phenomena in some disease vector species [30]. The indiscriminate use of drugs and the pervasive practice of self-medication in developing countries have contributed to the widespread occurrence of drug resistance in some parasite populations, as is the case of malaria in parts of Africa [31] and Southeast Asia [32].

#### Combined health determinants and their health outcomes

Extrinsic and intrinsic determinants of communicable disease transmission will often synergize in a negative way when clustered together. Deficient diets leading to immune deficiencies and lack of nutrients in high-risk (neglected) populations will lead to malnutrition and to an increase in the susceptibility to human pathogens. Severe hunger in poor children is also associated with chronic illnesses, behavioral problems (high anxiety and stress) [33] and learning disabilities [34]. Lack of access to health services will result in the deterioration of a person's health status, thus further hampering his or her productivity as a member of the work force and within the family (added burden to the family). Malnutrition [35], diarrhea, anemia and other complications of soil-transmitted helminth infections will often lead to growth stunting [36], school absenteeism [37] and also affect a child's ability to learn [38], reducing his or her chances of a better-paid and safer job later in life. Untreated parasitic skin infestations or infections (e.g., tungiasis and scabies often with secondary bacterial infection; and cutaneous larva migrans) [39] can lead to school absenteeism and lost work days. Poverty, poor housing, high population densities and unsafe or inadequate living conditions, combined with environmental conditions favoring vector breeding will readily promote the spread of some communicable diseases and trigger outbreaks in poor communities [40].

Many NDs such as soil-transmitted helminthiasis, schistosomiasis, or trachoma tend to *cluster*, both geographically and socially, in poor communities, neighborhoods and families, as do other communicable diseases such as malaria. Furthermore, only a small number of persons in a community will have high intensity infections (i.e., soil-transmitted helminth and schistosome infections tend to aggregate, that is, worm burden is concentrated in a small proportion of individuals in any community) [41,42]. In principle, knowledge of these phenomena can facilitate their prevention and control at the community level.

### B. Neglected populations and the need for an inter-sectoral approach

#### Beyond the health sector

Many determinants, especially the environmental determinants of disease, injury and death in developing countries lie *outside *the purview of the health sector. These determinants include poor living conditions such as unsafe drinking water, inadequate sanitation and excreta disposal, poor drainage, inadequate solid waste removal, poor housing, and indoor air pollution. The policies of the sectors that exert influence on these negative health impacts are usually not established according to public health criteria. Consequently, addressing comprehensive and sustainable solutions to these health problems cannot be the sole responsibility of the health sector.

Reducing risk factors needs to go hand in hand with the adoption of protective factors, including better access to services (health, environment, and education) and employment opportunities, backed by political commitment to guarantee their sustainability.

This situation calls for "thinking outside the box" (beyond the health sector) by incorporating an *inter-sectoral approach *[13,14,19,43], one that addresses the multiplicity of risk and protective factors and proposes strategies relying on synergies with other public health interventions (e.g., school deworming and nutritional programs), inter-programmatic synergies (e.g., IVM), articulations with sustainable development-based programs (e.g., schistosomiasis control and aquaculture micro-enterprise), and/or partnerships encompassing a wider set of stakeholders (e.g., Global Fund to Fight AIDS, Tuberculosis, and Malaria; Global Environmental Facility) [19].

Inter-sectoral approaches have been applied down to the community level for the control of infectious diseases and nutritional problems [44]. Furthermore, there are examples throughout the Americas that show that integrated, inter-sectoral work is being successfully conducted at the community level. For example, *integrated risk factor analysis *combining parasitological, environmental, social and economic risk factors in various communities to select foci for lymphatic filariasis mass treatment is used in the *favelas *(highly-impoverished urban communities) of Sanitary District II of the Lymphatic Filariasis Control Program called "*Xo Filariase*! [Shoo, Filariasis!] " in Recife, Brazil (personal communications, SA with T. Maciel Lyra, SMS/Recife, 2003–2004). Increasing the knowledge of protective and risk determinants and how they interact with each other in communities particularly exposed to NDs will help us tailor the interventions and find common ground with other sectors in order to achieve control or elimination of these infections.

### Justification

#### A. Why pursue integrated, multi-disease, inter-programmatic, and/or inter-sectoral, approaches for neglected diseases control and elimination?

1 Integrated, multi-disease, inter-programmatic, and/or inter-sectoral approaches give *added value *to disease control and elimination interventions. For example, the use of albendazole in lymphatic filariasis-endemic areas provides the added value of controlling soil-transmitted helminths [45]. Promoting the use of insecticide-treated bed nets or curtains helps interrupt the transmission of malaria and other vector-borne diseases in lymphatic filariasis foci [46]. Ivermectin is used to treat lymphatic filariasis and onchocerciasis. It has additional benefits against soil-transmitted helminths and head lice [47].

2 Integrated, multi-disease interventions are *cost-effective *by articulating one disease control intervention into another one, for example soil-transmitted helminthiasis and schistosomiasis control using combined therapies with albendazole and praziquantel [38]; or soil-transmitted helminth control combined with lymphatic filariasis elimination using albendazole plus diethylcarbamazine (DEC) [48].

3 We can *spread several benefits to the community with the same intervention*; an initiative that would surely be welcome by the community. For example, improved housing can protect against contact with some Chagas disease vectors and simultaneously reduce the risk of developing acute respiratory infections (ARI), thus improving the family's quality of life and perhaps even the market value of the dwelling.

4 *Inter-sectoral *interventions have positive impacts on family health and economic security, environmental sanitation, and even income generation, all of which are important to families and the community at large. Such interventions, when targeted to the more vulnerable or neglected groups, also assist in reducing health inequalities, an important new issue for many health agencies.

5 This proposed strategy also supports the *UN Millennium Development Goals *(MDGs) including 10 out of the 18 Millennium Declaration targets. Deworming cost-effectively improves nutritional status of poor children and communities, contributing to the goal of *Eradication of Hunger (MDG-1) *[49]. Deworming can improve school attendance [37,50] and thus increases the chances of completing primary education successfully (MDG-2, *Primary Education*). Promoting income-generating activities (e.g. micro-enterprises [51] for poor women) and educating impoverished mothers in child care contribute to the *Empowerment of Women *(MDG-3). Reducing the burden of parasitic diseases contributes to the *Reduction of Child Mortality *(MDG-4). Controlling iron deficiency and anemia due to hookworm [52] results in *Improvement of Maternal Health *(MDG-5). Combat of NDs [53] such as the intestinal helminth infections, leishmaniasis, parasitic skin diseases, and Chagas disease contributes to the goal of *Combat HIV, Malaria and other Diseases *(MDG-6). Implementing environmental sanitation reduces fecal contamination of groundwater and surface waters, and thus contributes to *Ensuring Environmental Sustainability *(MDG-7). An inter-sectoral approach to NDs prevention and control with a sustainable development focus involves establishing extended partnerships, compatible with the goal of *Global Partnerships for Development *(MDG-8) [54].

#### B.What opportunities and entry points are there for integrated, multi-disease, inter-programmatic, and/or inter-sectoral approaches?

The strategy proposed here focuses on a set of *micro-level interventions *(community, family, individual) to manage selected extrinsic determinants of NDs through integrated, multi-disease, inter-programmatic, and/or inter-sectoral approaches including improved health services, environmental sanitation and improved housing, better access to foods and key micronutrients, educational access for children and women, community participation and micro-enterprise development (e.g., urban and rural household-level food production). However, there is no universal recipe or protocol to manage these determinants. A specific strategy will have to be tailored to the local conditions, partners and resources available in each community or area. General examples of the opportunities and entry points to manage the determinants are elaborated below. Macro-level (policy) interventions are beyond the scope of this analysis and are dealt with elsewhere.

##### Health services

Management of morbidity due to leprosy is well integrated in health services in most leprosy-endemic countries, and could easily accommodate a lymphatic filariasis morbidity component. Combined mass drug administration (MDA) for lymphatic filariasis elimination and soil-transmitted helminth control is recommended by WHO. Soil-transmitted helminth and schistosomiasis control may be articulated with integrated management of child and adolescent health and development strategies and their syndromic approach to disease control. Where reduction of maternal mortality and improvement of women's health is the core of a country's strategic health plan hookworm control can be an integral part of such a plan. Combined MDA for schistosomiasis and soil-transmitted helminth control has been recommended by WHO for school-age children at high risk in areas of their geographic overlap [55]. Soil-transmitted helminthiasis and schistosomiasis control can, in principle, be combined with lymphatic filariasis elimination [38] in areas where the three disease groups overlap as in some coastal areas of NE Brazil.

Public health interventions can also be integrated along *other *sectors such as:

##### Environment (environmental sanitation, environmental quality & environmental management)

Lack of potable water supply, sanitation (especially excreta disposal), lack of solid waste collection and disposal and household cleanliness, and lack of animal control have all been identified as important risk factors in the transmission of diarrheal diseases, intestinal parasites, and skin diseases [56], and lack of household and yard tidiness with the presence of dengue vectors [57]. Multi-sectoral interventions in these areas have resulted in major reductions in the incidence and prevalence of some NDs and mortality in infants and young children in poor communities [58]. A recent systematic review and meta-analysis presents the positive impacts of water supply and hygiene interventions in reducing diarrhea in less-developed countries [59]. Examples of innovative interventions which address the disease determinants include home-based water treatment systems with filtration, flocculation and safe sealed storage containers [60]; simplified or condominial sewerage for shantytowns and other areas of urban poverty [61] and ecological disposal of excreta by the separation of urine and feces [62]; promotion of manual sanitary landfills; improved household and neighborhood drainage systems [63]; education to promote household hygiene, cleanliness and tidiness [64]; and animal corralling [65]. Improvements in rural housing (floors, ceilings, walls, and windows) have reduced the transmission of Chagas disease [66], while the use of mesh screens (for eaves, windows and doors) and sealing eaves have reduced human exposure to malaria vectors [67]. Domestic fly and mosquito control can be implemented in the household by use of deltamethrine-impregnated curtains [68]. Urban drainage improvements and improved household water storage systems can reduce urban malaria [69]. Improved wood stoves (biofuel stoves), substitution of biofuels with gas or other improved rural fuel/energy sources, and improved kitchen ventilation all reduce exposure of women and young children to harmful indoor air pollutants arising from exposure to smoke from traditional woodstoves and open fires [70]. Solar stoves, solar water disinfection and solar energy panels are possible alternatives to traditional wood stoves, boiling water with biofuels and burning kerosene lamps in the home, though their higher costs must be addressed [71]. Encouraging urban reforestation with local fast-growing trees and planting bamboo on the steep slopes where shanty towns are located will reduce soil erosion and vulnerability to small landslides [72]. The presence or absence of several of the interventions mentioned here is proposed as useful indicators of exposure to environmental health risks in developing countries [73].

#### 3 Education and School Health

School deworming programs (health education and treatment interventions) have been successfully incorporated into school health programs [74] and can be part of Healthy Schools initiatives. They can also be part of other community-based sustainable development initiatives which, for example, combine improved water supply, safe excreta disposal and hygiene education interventions for the school children [75]. The FRESH initiative (Focusing Resources on Effective School Health) is a model of an integrated approach to improving the health of school children; it focuses on four key components – health-related school policies, provision of safe water and sanitation, skills-based health education, and school-based health and nutrition services (including deworming and addressing micronutrient deficiencies) [76]. Lack of hygienic behavior by children clearly increases the risk of diarrheal diseases [77] and soil-transmitted helminthiasis [78]. Programs which educate women and children about the importance of proper handwashing with soap or ash and clean water can reduce diarrheal diseases incidence, acute lower respiratory infections, impetigo and soil-transmitted helminth infections in children of poor communities [59,79,80]. Nutrition education for women focused on basic nutritional concepts has a positive impact on the nutrition of their own children as well as the children of her neighbors [81]. Deworming programs have even been combined with family planning education programs in the Philippines [82].

#### 4 Nutrition and Food Security

Improved nutritional status is one necessary element of food security for the individual and the family. Treatment of intestinal worms enhances the value of food and micronutrient delivery programs [83]; anti-helminth treatment helps control iron deficiency anemia and its sequelae (including cognitive impairment). Deworming activities can be combined with vitamin A supplementation and the trachoma SAFE intervention (Surgery, Antibiotic Therapy, Facial Cleanliness and Environmental Improvement). Addressing key micronutrient deficits [84] (e.g., zinc deficiency which is causally associated with diarrhea, pneumonia and malaria in children under age 5 [85]) can be done by adding micronutrients to key foods in the local diet or to condiments such as table salt [86]. In areas endemic for lymphatic filariasis, DEC is added to table salt for mass treatment of the disease and can eliminate transmission within one to two years. In principle, DEC-salt can be combined with iodine and fluoride; Haiti has conducted a successful pilot project fortifying salt with DEC and iodine. Nutrition, deworming and family planning efforts have been integrated in a project in Sri Lanka [87]. Where hunger and undernutrition are addressed by supplemental feeding, school feeding programs, and other nutritional interventions in poor communities [88], some pilot projects have successfully combined deworming and supplemental nutrition in Africa, Asia and the Americas through partnerships involving non-governmental organizations (NGOs) and international agencies such as WHO, UNICEF and the World Food Program [89,90].

#### 5 Economic Development

Cooperatives can supply and manage insecticide-treated bednets or curtains [91] to the community and generate income. For example, in Leogane, Haiti, the NGO "KOLEMO" (Komite de Leyogan Pou Moustik) has developed a successful microlending program for local seamstresses affected by lymphatic filariasis to stitch mosquito bednets, which are then treated with the pyrethroid deltramethrin by the NGO and sold by the seamstresses to the community for a small profit which pays their labor. Use of the bednets reduces the number of bites by local mosquitoes that transmit lymphatic filariasis and malaria. Another cooperative program in Leogane, Haiti has provided nutritional rehabilitation of malnourished children [92], and improved employment and income distribution through innovative interventions such as local-level micro-financing and micro-credit schemes; these have also worked in Bangladesh and elsewhere [93]. Sustainable rural development projects (as promoted by the Heifer Project International, World Resources Institute, World Wildlife Fund, Nature Conservancy, International Union for the Conservation of Nature, etc.) which have integrated "better life programs" [94] and micro-credit projects [95] provide opportunities for rural and urban income generation and can also provide windows of opportunity for education in the prevention and control of NDs.

#### 6 Urban Improvement and Renewal

Well-planned urban improvement and renewal projects can improve community health and safety [96,97] (e.g., improved housing and street lighting; reducing environmental lead exposure [98]; more parks and green areas for recreation [99], safe bikeways and pedestrian paths [100]) and other integrated urban planning approaches such as integrated water basin development in Indonesia [101]. Integrated planning of urban improvement programs where the public health agency participates fully with other city agencies (public works, environment, and social services) [102] have proven successful in Cuba (e.g., *Movimiento de Municipios Productivos *[103]). Local public health authorities can also promote the use of a new public health tool, health impact assessment (HIA) [104], for urban development and urban renewal projects [105], thus helping prevent disease problems.

#### 7 Agriculture (Family and Community Agriculture, Horticulture and Agroforestry; Small Animal Husbandry)

Home gardens, community gardens and home-based small animal husbandry [106] and aquaculture [107] may be promoted to address income generation, nutrition and health needs simultaneously [108]. At home families can produce fruits, vegetables, nuts and medicinal plants in home gardens and even in small containers. Family food production also can help address the serious risks of food insecurity in poor communities though further study is needed [109]. Successful urban agriculture models exist in East Africa [110] and elsewhere. Local neighborhood cooperatives can supply safe compost for use in urban homes and community gardens. It can also be sold. Planting selected fruit trees at home can help augment the dietary intake of vitamin A [108], vitamin C and perhaps other micronutrients hopefully without need for pesticide application. As an example, the hearty shrub acerola or Barbados cherry (*Malphighia glabra*) is widely grown in home gardens in the Caribbean, Mexico, and Central and South America and contains high levels of vitamin C; it is propagated readily from seeds or cuttings. Selected local fruit and nut trees can also be planted in public places with free access as seen with avocado and jack fruit trees in Brasilia, Brazil and walnut trees in Yolo County, California. Planting fruit and nut trees could be targeted to areas in or near highly-impoverished communities. Small-scale animal husbandry (urban or rural) can increase women's and family income [111]. Small-scale animal husbandry is also an opportunity for veterinarians, public health specialists, educators and community members to work together to educate poor communities about preventing and controlling local parasitic and zoonotic diseases (e.g., taeniasis, toxocariasis, toxoplasmosis, tungiasis, rabies and leishmaniasis) through animal health care and vaccination, animal control and/or corralling.

#### 8 Primary Environmental Care (PEC)

Primary Environmental Care is an integrated and *participatory *approach to analyzing and solving community environmental health problems which was developed in the 1990s by UNICEF and PAHO/WHO [112]. The PEC approach is based on cooperation and partnerships between different stakeholders to identify key problems and develop sustainable solutions, and relies on active community participation [113]. For example, PEC is a tool that can in principle promote safer and more hygienic local environments and community responsibility, such as the corralling and control of pigs [114], poultry [65] and livestock and stray dogs [115] in the community. PEC could also help address problems such as family and neighborhood exposure to indoor air pollutants from biofuel use that contribute to the risk of ARI especially in women who work all day at home and house-bound young children [73,116].

#### 9 Promotion of Tourism

Tourism, both domestic and international, is a key part of the economy of many countries (e.g., Haiti's economy once depended strongly on tourism; Honduras' economy today continues to rely on tourism), cities and towns. However, the concerns of the tourist sector about communicable diseases and food safety [117] can stifle investment and growth of this sector. Integrated public health interventions focusing on improving environmental and sanitary conditions and hygiene behaviors in public markets and tourist hotels could help generate more tourism and income while reducing the occurrence of certain communicable and NDs (e.g., infestations of rodents associated with leptospirosis transmission, and insect vectors associated with dengue, leishmaniasis, malaria or lymphatic filariasis transmission). Food safety programs for tourist restaurants, hotels, street vendors and public markets are being promoted in some countries to address food-borne parasitic and microbial infections and travelers' diarrhea [118].

#### Three scenarios

We have developed three hypothetical scenarios to illustrate how an integrated, multi-disease, inter-programmatic, and/or inter-sectoral approach could be applied in three very different populations (an impoverished periurban community, an indigenous community, and a medium-sized city with a mix of populations).

The city of Jaboatão dos Guararapes on the coast of Pernambuco state, Brazil has a *favela *of about 40,000 residents including many children, mostly poor and undernourished migrants from the dry interior of the state seeking employment in the urban area (i.e., environmental and economic refugees). The *favela*, located beside a large shallow lake (Lago do Naútico) receiving untreated sewage from two cities, is endemic for lymphatic filariasis, schistosomaisis (*Schistosoma mansoni*) and soil- transmitted helminthiasis among other NDs and experiences domestic fly infestations. The *favela *has no potable water system or sewerage, no solid waste collection, and few latrines. Drinking water is trucked in and sold by private vendors. Shallow surface drainage canals cross the community, and are choked with weeds and trash and harbor the intermediate host snail of *S. mansoni*. The lake, used for fishing, recreation and washing clothes and dishes, floods the community periodically thus spreading excreta widely and it also harbors the snail intermediate hosts of *S. mansoni*.

In a multi-staged approach to prevention and control in this *favela*, the most critical interventions to improve the health of this community with respect to these NDs would be regular chemotherapy using a benzimidazole drug (albendazole or mebendazole) for soil-transmitted helminth infections and praziquantel against *S. mansoni *infections. If the community prevalence or individual worm burdens are high enough, MDA would be indicated to reduce worm burdens and accompanying morbidity in children, adolescents and adults. Community health education and appropriate social marketing of targeted health messages would be part of these first-stage interventions. In a second stage of interventions (medium- and long-term), the community would reap significant health benefits from a series of infrastructure interventions especially provision of safe excreta disposal and safe community drinking water supplies, improved surface-water drainage to free blocked canals, frequent solid waste collection and secure disposal, each accompanied by health and environmental education and social marketing. To aid in the sustainability of these interventions through community participation, improved opportunities for local employment could be encouraged through training and organization of the existing trash scavengers who already operate their own waste-separation (recycling) sites. Primary environmental care may play a useful role in promoting waste management and alleviating bad surface drainage, through community-based actions. Women's cooperatives and micro-credit programs could be promoted, with a health focus or with health improvement as an expected outcome. Community small-scale horticulture (home gardens) and animal husbandry (e.g., poultry) could help address chronic undernutrition especially in the community's children. In summary, the management actions to control the major extrinsic determinants of disease in this impoverished community in Jaboatão dos Guararapes must be undertaken not only by the health sector but other sectors as well [13,14].

The Yanomani communities of northern Brazil (states of Roraima and Amazonas) are a migratory indigenous population, who live in an onchocerciasis-endemic area and number about 10,000 people. Several families (often 30–100 people) and their dogs usually live in large communal houses or lodges called *malocas *without interior walls. Malnourished young children are not uncommon, while trachoma and tuberculosis are reported from the Yanomami. The communities also endure high burdens of soil-transmitted helminths and the ectoparasitic flea *Tunga penetrans *which causes severe disability of hands and feet. Tungiasis is also present in their dogs. An on-going indigenous health care intervention, in which MDA with ivermectin is provided to the Yanomami communities twice a year for onchocerciasis elimination, is expected to have a beneficial effect by also reducing the soil-transmitted helminths in children and adolescents, though this has not been measured as yet. Topical ivermectin application can be used to reduce the lesions caused by *T. penetrans *fleas [119]. Ivermectin (in a different dosage) is also used to treat tungiasis in dogs, and could be delivered to their pets during the twice-yearly mass treatment rounds for onchocerciasis elimination. Micronutrient supplements such as vitamin A and trachoma screening with antibiotic treatment could be provided to the most vulnerable groups, typically children and adolescent and pregnant women, during the course of the ivermectin mass treatments to eliminate onchocerciasis. Tuberculosis and leprosy screening could also be carried out during MDA. The communities seldom have a safe water source or safe excreta disposal systems. Water is usually taken directly from streams and open defecation is the norm. If accepted by the communities, health education on hygiene behavior (especially hand hygiene), simple home-based water treatment systems (discussed above) and/or improved environmental sanitation (e.g., mixing feces with wood-fire ash or lime and shallow burial, or perhaps dual-chamber ventilated improved pit (VIP) latrines made of local materials) could reduce the worm burden. Improved safe water with hand hygiene could reduce trachoma, though any increase in water use inside the *maloca *would need to be accompanied by wastewater management. Smoke from wood fires used in the *malocas *probably contributes to the burden of upper respiratory diseases seen in the Yanomami communities, especially children. Pilot projects for improved housing ventilation and perhaps the introduction of efficient biofuel ovens/stoves could be of interest to the communities, and incorporated into their health or environmental education programs. For communities which return to their villages each year, there may be a role for introducing the cultivation of perennial fruit trees if not home gardens.

**Figure 1:**
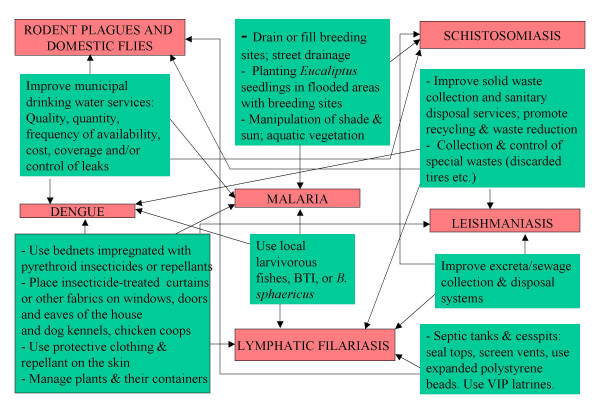
Multi-disease interventions for the prevention and control of vectors in urban areas – conceptual model.

In a perhaps typical example of the complexity of urban communicable diseases and NDs ecology, the city of Imperatriz in western Maranhão state, NE Brazil has a diverse population of over 230,000 residents including impoverished areas, squatter settlements, and river traders and fishing communities, and is located along the banks of a large river (River Tocantins) which brings annual flooding of parts of the city. The city experiences transmission of malaria along the riverside, and leishmaniasis, leptospirosis and dengue cases in the city, among other communicable diseases. Figure 1 is a model which illustrates sets of possible vector-borne disease interventions for cities (involving several municipal services sectors) to manage certain determinants of these diseases simultaneously and synergistically. This model of specific environmental interventions uses a simple systems diagram, which could be adapted for other specific communicable disease and NDs control contexts. The sets of interventions are shown in green with arrows from each intervention leading to two or more parasitic or vector-borne diseases (in red), indicating that the set of interventions can *in principle *be used in an integrated manner against several diseases (or their vectors) in the same geographic areas. (Not *every *intervention in the box can be used against every disease or vector linked by the arrows; similar interventions are clumped together to make the model more illustrative.) The model's true application in the field will depend on the array of local vectors, diseases, environmental conditions, health and environmental services, and epidemiologically-important human behaviors in specific populations.

In reading the illustrative model, for example, the interventions by the sector responsible for improving municipal drinking water services (in terms of quality, quantity, frequency of availability, cost, coverage and/or control of leaks) can be protective against dengue vectors, urban and periurban schistosomiasis, and even domestic rodents and flies which often prefer moist environments (such as those created by leaking water pipes and sewerage pipes). In another example, the municipal sector responsible for solid waste management can improve solid waste collection and sanitary disposal services; promote recycling and waste reduction, and collection and control of special wastes (discarded tires etc.) which can have an impact on the vectors of dengue (when breeding in discarded tires or other discarded water-holding wastes), leptospirosis and domestic fly plagues (by timely removal and disposal of discarded foods in garbage which act as food sources for domestic rodents and flies), and lymphatic filariasis (where uncollected solid waste on the streets blocks and pools urban surface drainage thus creating better conditions for *Culex quinquefasciatus *vector breeding).

## Summary

• Neglected populations living in poverty throughout the developing world tend to be extremely burdened with a series of neglected communicable diseases and often marginalized by the health sector.

• In order to successfully strengthen the public health agenda for NDs in neglected populations, it is important to consider the contextual determinants of health. These are both intrinsic and extrinsic to human populations. The combination of both determines the epidemiological pattern of communicable diseases (disease spectrum) at the local level and help identify appropriate interventions.

• Integrated, multi-disease, inter-programmatic, and/or inter-sectoral approaches give added value to disease control and elimination interventions, as have been demonstrated in various countries.

• We have argued for a new way to think about addressing the problems of NDs in neglected populations, using integrated, multi-disease, inter-programmatic, and/or inter-sectoral approaches. Numerous examples of specific successful interventions to manage disease determinants in various types of communities have been presented, but few studies exist where such measures were applied in an integrated and coordinated manner.

• Three hypothetical scenarios have been developed here to illustrate how multiple interventions could manage multiple disease problems through integrated, multi-disease, inter-programmatic, and/or inter-sectoral approaches in three very different populations (an impoverished periurban *favela*, an indigenous community, and a medium-sized city with a mix of populations and diseases).

• It is our aim that this review and analysis will stimulate intra- and inter-sectoral dialogue which will help in the construction of new NDs strategies (particularly for the parasitic diseases) which could benefit the poor and marginalized based on the principle of sustainability and understanding of some key determinants of health, and lead to the establishment of pilot projects and activities which can contribute to the MDGs.

## List of Abbreviations

ARI. Acute respiratory infections.

BTI. *Bacillus thuringiensis *ssp. *israelensis*

DEC. Diethylcarbamazine.

FRESH. Focusing Resources on Effective School Health

HIA. Health impact assessment.

HIV/AIDS. Human Immunodeficiency Virus/Acquired Immunodeficiency Syndrome.

IVM. Integrated Vector Management.

MDA. Mass drug administration.

MDGs. Millennium Development Goals.

NDs. Neglected diseases.

NGO. Non-governmental Organization.

PAHO. Pan American Health Organization.

PEC. Primary Environmental Care.

SAFE. Surgery, Antibiotics, Face-washing and Environmental improvement.

UNICEF. United Nations International Children's Emergency Fund.

VIP. Ventilated improved pit

WHO. World Health Organization.

## Competing interests

The author(s) declare that though they are employees of the Pan American Health Organization and the World Health Organization, the contents of this paper are the sole responsibility of its authors and should not be construed as speaking for the policies of the Pan American Health Organization and the World Health Organization. This paper is a tool towards further scientific dialogue and discussion among public health professionals.

## Authors' contributions

JE conceived the idea of the paper, wrote the first draft, and is the principle conceptual author. SA was responsible for additional concepts, for gathering the majority of the supporting evidence, examples, and for creating the three scenarios. SA developed Figure 1. Both authors read and approved the final manuscript.

## Pre-publication history

The pre-publication history for this paper can be accessed here:

http://www.biomedcentral.com/1471-2458/5/119/prepub
